# Team-based learning (TBL) in the medical curriculum: better than PBL?

**DOI:** 10.1186/s12909-017-1068-z

**Published:** 2017-12-08

**Authors:** Annette Burgess, Jane Bleasel, Inam Haq, Chris Roberts, Roger Garsia, Tomas Robertson, Craig Mellis

**Affiliations:** 1 Education Office, Sydney Medical School, Edward Ford Building A27, University of Sydney, Sydney, 2006 Australia; 2 Sydney Medical School – Northern, University of Sydney, Sydney, Australia; 3 Sydney Medical School – Central, University of Sydney, Sydney, Australia; 4 Clinical Immunologist, Department of Immunology, Royal Prince Alfred Hospital, Camperdown, 2050 Australia; 5 Central Clinical School ( RPAH campus level 4), Sydney Medical School, University of Sydney, level 2 Medical Foundation Building Parramatta Rd, Camperdown, NSW 2050 Australia

## Abstract

**Background:**

Internationally, medical schools have long used a variety of approaches to develop hybrid Problem based learning (PBL) curricula. However, Team-based learning (TBL), has gained recent popularity in medical education. TBL maintains the advantages of small group teaching and learning, but in contrast to Problem-based learning (PBL), does not require large numbers of tutors. In 2016, TBL was introduced to Year 1 of the Sydney Medical Program (SMP).This study sought to compare students’ perceptions of using TBL in place of PBL.

**Methods:**

Year 1 students (*n* = 169) completed three PBL and three TBL sessions during one of the following teaching blocks: Musculoskeletal (*n* = 56), Respiratory (*n* = 59) or Cardiovascular (*n* = 54). Student feedback following completion of each block of teaching was collected by questionnaire, using closed and open ended items. Data were analysed using descriptive statistics and thematic analysis.

**Results:**

In total, 144/169 (85%) of participants completed a questionnaire regarding PBL, and 152/169 (90%) completed a similar questionnaire regarding TBL. The students found positive aspects of their TBL experience to include the smaller group size, the use of readiness assurance tests, immediate feedback from senior clinicians, and time efficiency. In PBL, students reported that variable expertise of tutors; limited direction; and large group size hindered their learning.

**Conclusions:**

Overwhelmingly, students preferred TBL over PBL, as the optimal teaching strategy. Students found the structure and format of the TBL sessions more conducive to learning, engagement and participation than PBL sessions. Although the use of TBL required an instructional approach, needing direction from the tutor, it remained student-centred, generating a range of positive outcomes. Study results provide confidence to change from PBL to TBL within Year 1 and Year 2 of the SMP in 2017.

## Background

Internationally, medical schools have long used a variety of approaches to develop hybrid Problem based learning (PBL) curricula [1]. However, over time, a number of these hybrid PBL models have become less effective, and decreasingly aligned with the intended student-centred learning philosophy [2]. With extensive face-to-face PBL group meeting time, yet limited individual accountability for contribution to group work, student satisfaction with hybrid PBL models has decreased in recent years [2]. Introduced to Sydney Medical School (SMS) in 1997, a hybrid PBL approach has provided a long-established form of teaching within the medical curricula. However, increasing student numbers (from 142 Year 1 students in 1997 to 332 in 2016) and limited teaching resources, have rendered this model of teaching unsustainable [3]. Dissatisfaction with PBL has arisen from increasingly larger groups of students (*n* = 10), the time intensiveness of PBL (two 1.5 h sessions each week), insufficient peer engagement, and the variable expertise, teaching experience and enthusiasm of facilitators [3]. Our students’ declining opinion of PBL, can be attributed to lack of standardisation across the cohort, and the increasing value placed on time efficient learning strategies [3]. Earlier studies similarly reported “haphazard” PBL tutorial processes, and lack of student accountability as key contributors to students’ dissatisfaction [4–6]. Excellence in medical education requires adaptation of the curriculum to meet student needs [7]. Adopting a blended learning approach, with appropriate instructional strategies and efficiencies, has the potential to enhance student engagement both inside and outside of the class room [8].

An alternative to PBL that adopts a blended learning approach, is Team-based learning (TBL), which has gained recent popularity in medical education [9]. TBL allows medical educators to provide students with resource effective, authentic experience of working in teams to solve real life clinical problems [10]. Our 2014 pilot study (*n* = 20) of TBL [3], indicated that students favoured many aspects of the TBL process, including the pre-class work, the in-class initial tests with immediate feedback, and the problem-solving activities. Students found the advantages of TBL over PBL included better engagement in learning, deeper understanding of concepts, and a sense of responsibility towards teammates [3]. However, negative aspects of the students’ TBL experience included limited time to complete problem-solving activities, and a de-emphasis on the student-centred approach involving clinical reasoning among student groups. In 2016, based on our previous TBL pilot experience, as well as wider literature evidencing the effectiveness of TBL in health education, we sought to incorporate a sustainable and standardised TBL model across the Musculoskeletal sciences, Respiratory sciences, and Cardiovascular sciences blocks of the Year 1 medical program. Key features of TBL principles were adopted, including appropriate allocation of individuals to groups, prescribed out-of-class preparation, pre-class individual and team tests, immediate feedback, and problem-solving activities with all team work within a single session [10].

In recognition of the large variance in implementation and reporting of TBL, within the diverse range of settings, content areas and learners within health sciences education, Haidet and colleagues (2012) recently proposed a set of guidelines for standardising the way in which TBL is reported and critiqued [10]. We used these guidelines to outline the scope of our TBL program, and report our implementation. According to Haidet (2012), the “seven core design elements that underlie the TBL method” are: 1) team formation, 2) readiness assurance (RA), 3) immediate feedback, 4) sequencing of in-class problem solving, 5) the four S’s (significant problem, same problem, specific choice, and simultaneous reporting), 6) incentive structure, and 7) peer review [10].

This study sought to report on our implementation of TBL in Year 1 of a graduate entry medical program during the 2016 Musculoskeletal Sciences block, Respiratory sciences block, and Cardiovascular Sciences block. Our aim was to explore students’ perceptions of their experience during TBL, drawing some comparisons with their experience of PBL sessions.

## Methods

### Sampling and participants

In 2016, 169 Year 1 students completed three PBL and three TBL sessions during one of the following teaching blocks: Musculoskeletal (*n* = 56), Respiratory (*n* = 59) or Cardiovascular (*n* = 54).

In total, 169 Year 1 students participated in the study. Convenience sampling was used to select 18 established PBL groups (six for each teaching block). In each teaching block, the same six PBL groups were combined to form one TBL class, consisting of nine or ten teams of five or six students.

### Content of the PBL and TBL sessions

The weekly learning topics for PBL and TBL during the Musculoskeletal Sciences, Respiratory sciences and Cardiovascular sciences are outlined in Table 1.

**Table 1:** weekly learning topics of Musculoskeletal sciences, Respiratory sciences and Cardiovascular sciences block

Week	Title	Topic
Musculoskeletal Sciences block		
Problem Based Learning		
Week 1	New wheels – fractured femur	Fractured femur (MVA)
Week 2	Not just a game	Acute knee injury
Week 3	I always work hard	Sciatica/back injury
Team Based Learning		
Week 4	An embarrassing fall	Fractured NOF & osteoporosis
Week 5	I must be getting old	Osteoarthritis
Week 6	Why me?	Rheumatoid arthritis
Respiratory Sciences block		
Problem Based Learning		
Week 1	Not at fault	Chest trauma, pneumothorax
Week 4	Ex-navy	Interstitial lung disease
Week 6	A different cause of cough	Cystic fibrosis
Week 7	Difficult circumstances	Pneumonia, Otitis media
Team Based Learning		
Week 2	Wheezing and breathless	Asthma
Week 3	A nasty cough	Acute exacerbation of chronic obstructive pulmonary disease
Week 5	Sleeping on the job	Sleep apnoea, respiratory failure
Cardiovascular Sciences block		
Problem Based Learning		
Week 3	Ms Newman’s indigestion	Myocardial ischaemia
Week 4	A breathless pregnancy	Valvular heart disease
Team Based Learning		
Week 2	Going down hill	Heart failure
Week 5	Jennifer and David’s baby	Congenital heart disease, Down sydnrome
Week 6	A sudden collapse	Syncope and arrhythmia/hypertension

### Structure of problem based learning

At the time of this study, Sydney Medical School (SMP) offered a four year graduate entry medical program, with a hybrid PBL curriculum. Students were assigned to PBL groups of ten students. During Year 1 and Year 2 of the program, students attended two 1.5 h weekly PBL tutorials on university campus on separate days. The first PBL session was student led, and a facilitator was present at the second PBL session. In collaboration with their group members, students were expected to analyse a clinical problem, formulate hypothesis, and undertake self-directed learning tasks between the two PBL sessions.

### Structure of team-based learning

The TBL sessions were held once per week for two hours, replacing the two PBL sessions that would normally occur.

#### Team formation

To reduce potential discrepancies in gender, international status, and science background of students, allocations of students into their TBL teams was carried out with a minimisation technique using a system of linear equations [11]. Students were allocated to teams consisting of either five or six students, and teams remained together for each teaching block. The Musculoskeletal class (*n* = 56) consisted of 10 teams of five students, and one team of six students; the Respiratory class (*n* = 59) consisted of seven teams of five students and four teams of six students; and the Cardiovascular (*n* = 54) class consisted of six teams of five students and four teams of six students.

#### Pre-class reading

Prior to class, students were allocated compulsory readings or/and pre-recorded lectures.

#### In-class schedule

The structure *during class* is outlined in Table 2, including the Individual Readiness Assurance Test (IRAT), Team Readiness Assurance Test (TRAT), Immediate feedback, and Clinical problem solving activities. While the principles of TBL were paramount in course design and implementation, we note that we did not include formal peer evaluation or a formal incentive structure. Rather, we assumed that students would be motivated by a sense of accountability to their teams to prepare and participate in the TBL readiness assurance process and problem-solving activities. We also note that we used open-ended responses during the problem solving activities, rather than a specific choice that is simultaneously disclosed by student teams. Although we did not have a formal appeals and dispute process, there was opportunity within the immediate feedback session for students to promote discussion and challenge answers.

**Table 2:** Activity schedule during Team-based learning sessions

Time	Activity	Explanation of activity
10 min	Individual Readiness Assurance Test (IRAT) (administered on paper)	At the beginning of each class, students’ individual knowledge of the pre-reading was assessed by 10 Multiple Choice Questions, using single best answer format, with five options.
20 min	Team Readiness Assurance Test (TRAT) (administered via laptop/Smartsparrow)	The same MCQ test was repeated by the students in their teams (TRAT), immediately upon completion of the IRAT. The test was administered online. One laptop per team was used, with the intent of promoting discussion to establish team consensus. For each question, teams who answered correctly on the first attempt received a score of 4, and those who answered correctly on the fifth attempt scored zero. These scores were then summed across the items to obtain a total score ranging from 0 to 40. Facilitators were able to view each team’s progress throughout the test, and at completion, all scores were made available to the entire class.
20 min	Immediate feedback from the facilitators	The correct answers were then released, and explained, giving immediate feedback on team and individual responses. Thereafter, the facilitator offered clarification, particularly where teams had experienced difficulty, or disputes.
60 min	Clinical problem solving activity	Students then worked in their teams on their problem solving activities, using knowledge consolidated through the prior steps.
10 min	Close	Key take home messages were summarised.

### TBL facilitators

Nine senior academic clinicians participated as facilitators: three Rheumatologists, three Respiratory physicians, and three Cardiologists.

### Data collection and analysis

#### Questionnaires

##### Student questionnaire

Two questionnaires, one regarding PBL, and one regarding TBL, were distributed to student participants following the completion of each teaching block (Musculoskeletal sciences, Respiratory sciences and Cardiovascular sciences). The questionnaires included closed items, (using five point likert-scale, with 1 being ‘strongly disagree’, and 5 being ‘strongly agree’) and open-ended questions. The questionnaire was adapted from a validated questionnaire designed by Thompson and colleagues (2009), to measure the quality of team processes in medical education [12]. Open-ended questions were also asked to elicit the best and worst features of the sessions.

Quantitative data were analysed using descriptive statistics. Thematic analysis was used to code and categorise qualitative data into themes. Once data had been coded and categorised into themes, the data within each theme were quantified in order to measure thematic prevalence [13].

### Ethics approval

The University of Sydney Human Research Ethics Committee approved the study. Written consent for participation was obtained from participants to enable us to include their data from this study.

## Results

### Questionnaire

#### Student questionnaire

In total, 144/169 (85%) of participants completed a questionnaire regarding their PBL experience, and 152/169 (90%) completed a questionnaire regarding their TBL experience as follows.

**Fig. 1:**
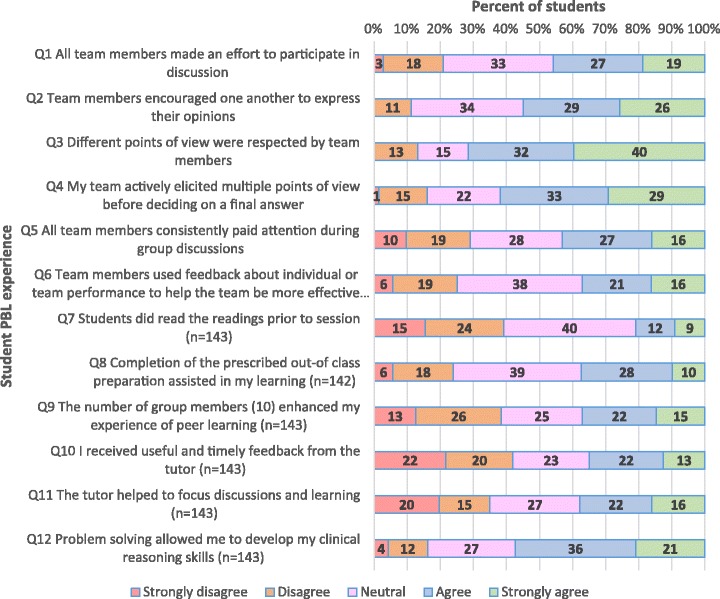
Student responses to closed items regarding experience in PBL (*N* = 144)

**Fig. 2:**
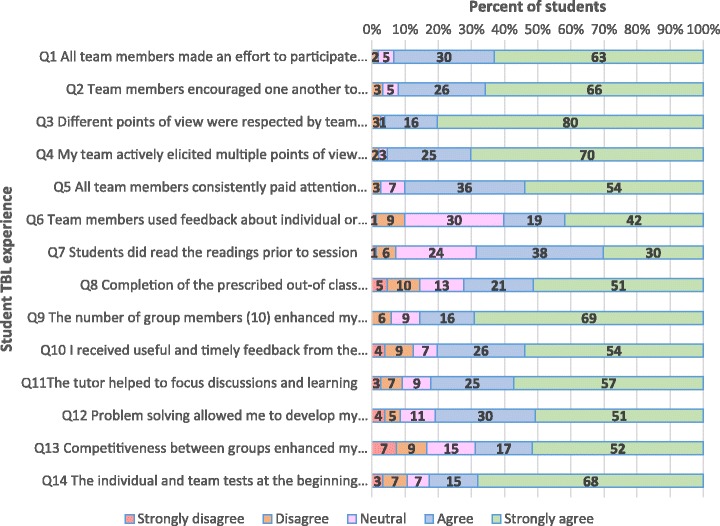
Student responses to closed items regarding their experience in TBL (*N* = 152)

**Fig. 3:**
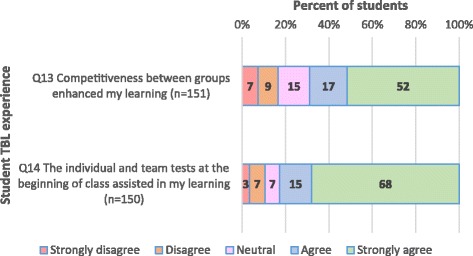
Student responses to closed items regarding their TBL experience (*N* = 152)

Student responses to closed items regarding their experience in PBL are shown in Fig. 1. Student responses to the same closed items regarding their experience in TBL are sown in Fig. 2. Responses to two additional questions specific to TBL are shown in Fig. 3. Overwhelmingly, students preferred the smaller group size of TBL, with 85% strongly agreeing or agreeing that in TBL *“the number of group members enhanced my experience of peer learning”*, compared to 37% in PBL (item 9). Notably, in TBL, 93% of students strongly agreed or agreed that *“all team members made an effort to participate in discussion”*, compared to 46% in PBL (item 1), which was consistent with other responses (to items 2 to 5) regarding team participation. Feedback was well facilitated in TBL, with 80% of students strongly agreeing or agreeing that “*I received useful and timely feedback from the tutor*”, compared to 46% in PBL (item 10). Feedback was also better utilised by students in TBL, with 61% strongly agreeing or agreeing that “*Team members used feedback about individual or team performance to help the team be more effective”,* compared to 37% in PBL (item 6). The tutors were better able to help “*focus discussions and learning”* in TBL (with 82% strongly agreeing or agreeing), than in PBL (with only 38% strongly agreeing or agreeing) (item 11). Importantly, students were generally satisfied with the problem solving activities within TBL, with, 81% strongly agreeing or agreeing that “*Problem solving allowed me to develop my clinical reasoning skills*”, compared to 57% in PBL (item 12). In TBL, 72% of students strongly agreed or agreed that “*Completion of the prescribed out-of class preparation assisted in my learning”,* compared to 38% in PBL (item 8).

Additionally, as shown in Fig. 3, the majority of students (83%) strongly agreed or agreed that in TBL, the *“individual and team tests at the beginning of class assisted in my learning”,* and 69% strongly agreed or agreed that the *“Competitiveness between groups enhanced my learning”.*

Responses to open ended questions regarding students’ perceived best and most difficult features of PBL are illustrated in Tables 3 and 4 respectively. The students found positive aspects of their TBL experience to include the smaller group size, the use of readiness assurance tests, immediate feedback from senior clinicians, and time efficiency. In PBL, students reported that variable experience of tutors; limited direction; and large group size hindered their learning.

**Table 3:** Students’ perceptions of PBL, including best and most difficult features (*N* = 144)

Theme	Examples of student comments	No. of similar responses
Most useful features of Problem Based Learning		
Discussion oriented sessions		61/144
Students enjoyed the discussion that took place within the PBL session, and the opportunity to teach and learn from each other	*PBLs are a chance to: meet and brainstorm ideas with peers, learn from peers and teach peers in a safe environment* *No time pressure – conducive for thorough discussion. Discussion and interaction with fellow students and being able to learn from each other*	
Clinical reasoning opportunity within PBL		23/144
Students liked the opportunity to discuss a clinical case with their peers, and learn from each others’ clinical experience Students found that working through detailed patient cases helped them to retain information	*Being able to recreate a clinical situation,* via *a PBL, is a great way to learn - especially in this block - as there is more correlation with the patients I have seen at hospital on clinical days. In addition, listening to various opinions of my peers gives me an insight into the way they think about the cases and process information.* *I like how detailed the patient histories are. When studying later on, it allows me to actually remember “Mr D’este has low back pain because he did X, and it resulted in Y and we treated him with A, B and C”. Getting a chance to discuss and work through a case with your peers.*	
Most difficult features of PBL and need for improvement		
Variable expertise and training of PBL tutors		78/144
Students perceive that the knowledge, engagement and experience of PBL tutors varies greatly Students felt their learning was dependent upon the PBL group allocation, which they perceived as unfair. Students felt it was necessary to have clinicians as tutors	*Tutor and students unable to validate information, we got into the habit of glossing over things because it would get too tedious* *Our PBL tutor, like the previous block, is not able to help us learn about different clinical cases, simply because they are not a clinician. It is unfair that some groups do actually learn more in these classes simply because they have better tutors.* *I found that the tutors who were non clinical based didn’t have much to say and weren’t very helpful when a question was targeted to them. I think tutors need to have a more relevant background in the framework of a case.* *There seems to be a lack of structure given to tutors that gives a disjointed feel to the PBLs. Prepare tutors in group facilitation or the subject matter*	
The PBL groups were too large		61/144
Students found that having 10 students per PBL made group work difficult Some students felt the ‘louder’ students tended to dominate discussion in PBL	*Group work is difficult with 10 people….means people can skip under the radar a little bit. With 10, there’s still a good 4–5 people that don’t participate strongly in discussion* *Too many people- I often didn’t feel comfortable talking and sometimes felt pushed over. We never really had a successful session- I actually feel I didn’t really learn much at all.*	
Inadequate direction and structure		59/144
Students found the PBL tutorials lacked guidance Students would like to have more direction regarding pre-readings to enable effective teamwork Students would like to have ensured they had completed tasks by the end of the PBL	*Sometimes we are not sure how deep we should know about certain topic, and questions in the students guide are too general/a bit vague*. The questions could be more specific *Not everyone comes with the same level of preparedness. More pre-work would be beneficial to increase confidence and understanding in the session* Produce a tangible document at the end which we need to submit so that I can take away and refer to in the future, rather than a vague memory of discussion.	
Inefficient use of time		28/144
Students felt their time was not used efficiently, and there was too much information to cover within the PBLs	*For my group, staying on time is very difficult. Some people in the group like to answer the discussion questions right down to the very last detail.* *Less time so there is more pressure for a concise discussion that is relevant.*	
Ineffective group dynamics		37/144
Students felt the group dynamic within PBL was hindered by lack of preparation, direction and feedback	*Power battles between people, no experts in the room and no certain answers to questions - if anything it creates more questions as opposed to answers* *Lack of universal participation (2–3 students), everyone is on their computers. Lack of engagement, lack of internal thinking due to obvious nature of the case study…. some members are content to watch or are intimidated by the bigger personalities.*	
Lack of prior knowledge and different		20/144
preparation requirements	*It was difficult to do independent research about topics that we are not familiar with.* *Going through a case without significant prior knowledge.*

**Table 4:** Students perceptions of Team-based learning, including best and most difficult features (*N* = 152)

Theme	Examples of student comments	No. of similar responses
Best features of Team-based learning		
Presence of Experts		89/152
Students found it valuable to have continual access to content experts as facilitators who would provide accurate information, feedback, promote and focus discussion, and reinforce knowledge They liked the immediate feedback that was continuously provided by the tutors following quizzes, and during the clinical problem solving activities Senior clinicians provided a clinical context	*Having multiple experts present to ask questions and explain the disease properly, really helped focus the discussions* *I liked that we have people at hand who really know their stuff instead of a volunteer with sometimes little knowledge of the topic as with PBL.* *Definitely the presence of tutors that specialised in the topic, because our questions can be properly answered. The session is also more efficient, tackling the most relevant aspects (clinical features and pathogenesis)* *Having the experts available in the room and giving us proper information and case studies and talking about their experiences in the clinic - much better than the tutors in PBL (worth the swap to TBL for this)*	
Readiness Assurance Process with discussion and feedback		98/152
The tests and feedback at the beginning of class helped to focus the session The tests helped students to reflect on their acquired knowledge and gaps in the knowledge Tests motivated students to prepare, and encouraged friendly competition between groups.	*Competition at the beginning of the lesson helped focus the discussion. It also ensured that everyone had the requisite knowledge to effectively participate in the TBL. Tests cause better focus. Experts gave definitive answers rather than questions.* *I really enjoyed the first hour quizzes and explanations since I learned a lot from those instead of just discussing amongst ourselves without knowing if we were headed in the right direction. The test at the beginning points out what you don’t know and then moving forward you know what to pay attention to.* *Quizzes to test learning/encourage people to do pre-work. MCQ as guide to what in pre-work was really important. Intergroup competition brings motivation.*	
Smaller Groups		51/152
Students found small groups of 5 to 6 students encouraged discussion within groups. Having multiple groups in one room also enriched the learning environment.	*The smaller group size effectively enhanced communication between team members and the condensed time frame ensured streamlined and focussed discussions.* *Smaller groups are better, helps discussion and discourages non-participation.Having multiple groups participate together also aided in learning.*	
Effective, structured and focussed format of the sessions		82/152
Students found the TBLs provided continuity to their learning, and provided an efficient method to revise and build on previous knowledge. Students found the structure of the TBL sessions helpful to direct their learning and increase participation Students found the structure of the TBL sessions reinforced and built on their prior learning.	*TBLs are phenomenal. They are as effective and useful as PBLs are ineffective and a waste of our time. TBL is more beneficial than 3 h of PBL so time effective. I like how it is only one session a week and that the key learning points are really emphasised.* *More focused, efficient but useful in assessing understanding and enhancing/reintroducing previous knowledge.* *Having a more structured discussion helped reduce time usually wasted in PBL. Shorter sessions equals less time in the week as contact hours – coupled with more high yield information. It was more concise than normal PBLs and the quizzes and pre-readings, really aided in learning. It feels like I learned more through TBLs than PBLs. The experts who were present at the sessions were very knowledgeable and helpful.* *The session is structured in a way that is conducive for recapping and reinforcing our prior knowledge in certain topics.*	
Pre-reading		28/152
Students found having set pre-reading helped students be on the same level, and aided collaboration	*Having a directed set of learning materials and readings helped the TBL to be a focused session increasing the yield of knowledge for the session…. meant we were more prepared to be effective straight away.*	
Most difficult features of Team-based learning		
Alignment of pre-readings		21/152
Students suggested the pre-readings should be more relevant to the test, with some compulsory, and some optional readings.	*Sometimes, I felt the readings weren’t as relevant to the questions in the test.* *More focused readings with more background optional readings.*	
Completion of all problem-solving activities		35/152
Many students indicated it was difficult to complete all of the problem-solving activities in the given time. Some suggested that an additional 15–30 min of class time would be helpful	*Perhaps make TBL an extra 15–30 min to ensure that the clinical problem has been resolved well enough.* *Working through the clinical problem solving in an hour can be tough but it is useful and I feel as though I understand it a lot better afterwards*	
Flow chart explanation		49/152
Students felt that further direction and discussion should be given around the flow-chart activity.	*Mechanism of the disease was challenging. More direction given with regards to the pathogenesis flowchart would help*	

## Discussion

This study sought to explore students’ and faculty’s perceptions of PBL and TBL during Year 1 of the medical curriculum, across the Musculoskeletal sciences, Respiratory sciences and Cardiovascular sciences blocks. Results indicate that students found their experience in TBL to be overwhelmingly more positive than their experience in PBL. Students reported working in small groups of five or six students, compared to larger groups of 10 in PBL, increased participation and peer learning. They found the readiness assurance process, including the individual and team tests, motivating and engaging; and immediate feedback from a clinical expert beneficial to their learning. However, students noted that during TBL they would have liked greater opportunity to discuss their completed pathophysiology flowcharts. Although students enjoyed opportunities for clinical reasoning and discussion in PBL, they found the variable experience of tutors, limited direction, and large group size hindered their learning.

Excellence in communication and team work is essential to health care and patient safety, particularly within increasingly complex healthcare systems [14]. The structure of TBL has elements conducive to preparing students to work in teams, synthesise evidence, and communicate with each other. Students commented that the smaller groups of TBL enabled greater participation, discussion and collaboration [15, 16]. Notably, in TBL, 93% of students strongly agreed or agreed that *“all team members made an effort to participate in discussion”*, compared to 46% in PBL. Designated preparation for essential knowledge acquisition for TBL shifted the burden of learning content during class [17]. The majority of students (83%) strongly agreed or agreed that in TBL, the *“individual and team tests at the beginning of class assisted in my learning”*. Additionally, the readiness assurance test provided the facilitator with a means to immediately assess student knowledge and understanding, and address specific needs [18]. Students noted they were more likely to come to class prepared in TBL, hence the quality of team and class discussion improved. Individual student accountability was fostered by the use of the individual assessment (iRAT), while the tRAT promoted effective teamwork. Students felt a sense of friendly competitiveness among teams, which enhanced motivation to prepare. Unlike in PBL, where individual students had different preparation requirements, in TBL, all students had the same pre-class requirements, and came to class ready to engage.

Teachers are expected to be experts in their fields, with the ability to guide students to take an active learning role [19]. By following the steps in TBL, three facilitators were readily able to manage 10 small groups of students (ie, 50 to 60 students) in one room. An advantage of TBL was the increased feedback and guidance from facilitators, who were content experts. In PBL, students found the variable expertise of their facilitators, and lack of guidance, led to uncertainty, and hindered learning progress within groups. Unfortunately, feedback was often lacking in PBL teaching sessions. However, provision of immediate feedback has the ability to enhance students’ understanding of their content knowledge [18], and is crucial to knowledge acquisition, application, and retention [20, 21]. Feedback was well facilitated in TBL, with 80% of students strongly agreeing or agreeing that *“I received useful and timely feedback from the tutor”* compared to 46% in PBL. Although it is widely accepted that the ideal PBL tutor is a group facilitator, rather than a content expert [22], without timely feedback, errors may go uncorrected, and a students’ sense of being “lost” with new content is amplified [21]. In TBL, faculty’s expertise is utilised to design a learning experience for students that is rich in feedback [23]. Students are never left in doubt regarding their understanding of the content, with feedback being received through the readiness assurance process, and during problem-solving activities, where facilitators assist individuals and teams as required. Additionally, the provision of clinical context within medical education helps students to understand and recall content [24, 25]. Students felt that provision of clinical examples and relevant scenarios by facilitators who were clinicians during TBL led to better engagement in learning, and an increased understanding of the relevance of the basic science concepts.

In TBL, the formal testing procedure, with the sequence of the readiness assurance process ensured students had several opportunities to engage with the content and gauge their own understanding [15]. Students built on their own learning by comparing their answers to other team members, and engaging in discussion in order to come to a consensus. The TBL format provides the opportunity for students to develop critical competencies relevant to health care education: teamwork abilities and critical thinking skills. [26] In PBL, our students reported such practices were limited, and hindered by large group sizes. A previous study reporting on the effect of group size in PBL in a dental school found a correlation between small (3) to medium (6) sized groups and increased satisfaction and self-directed learning among students, compared to larger groups (9) [27]. Team learning in both PBL and TBL is promoted through implementation of the problem-solving activities [28]. However, during TBL, the small size of groups meant that all students were forced to contribute to the problem-solving activities [29], and engage with the content. Completion of tasks required productive team interaction. Students felt that in TBL, unlike in PBL, having an end product, such as the drawing of a flowchart provided a sense of satisfaction for their teams.

Notably, key features favoured by students in PBL, were the discussion oriented sessions, with broader opportunities for clinical reasoning, and the opportunity for students to discuss their own clinical experiences. Previous literature has highlighted the advantage of PBL over TBL as being the small group discussion prior to self-study [30]. Evidence suggest that group discussion, where prior knowledge is activated, may have a positive impact on learning [31]. This is an element that could potentially be incorporated to improve our TBL sessions. Within the structure of PBL, students are encouraged to plan and monitor their own learning. In PBL, students are not given pre-reading assignments. However, they are encouraged to generate their own questions and issues for further self-directed learning and group discussion, promoting lifelong learning skills [30]. As Dolmans et al. [30] suggests, by combining the positive elements of PBL with TBL, student learning may be optimised.

### Study limitations

It is possible that students simply found the new method of teaching (TBL) to be novel, which may have made their responses more positive than if the study was carried out over a greater length of time. However, given the strength of students’ views, and the breadth of the study, across three blocks of teaching, we feel our conclusions and recommendations are accurate. Our findings may or may not be generalizable to other institutions and students.

Consistent with recent literature, and as noted by Michaelsen himself [32], application of TBL within the health care field is constrained by a number of pre-determined contextual factors, which reduce the ability to adhere to all classic design elements of TBL. We found allowing only one “specific choice” within the problem-solving activity phase of TBL “restricts the discussion to predetermined outcomes” [33], and instead used open-ended questions. However, the advantage of avoiding “Specific choice” within the problem-solving activity is that faculty do not need to generate the specific choice questions/answers (MCQs), which can be difficult and time consuming to write [33, 34].

## Conclusions

Increasingly, the practice of medicine is both team-orientated, and inter-professional, requiring co-ordinated efforts from a number of disciplines to provide the best outcomes for patients [23]. Changes in both curricula and pedagogy are needed to prepare students for demands of the increasingly complex healthcare systems. Graduate competencies have shifted from knowledge to the ability to solve complex problems, communicate and collaborate effectively. The students found positive aspects of their TBL experience included the smaller group size, the use of readiness assurance tests, immediate feedback from senior clinicians, and time efficiency. Additionally, the application of TBL principles meant the sessions were not reliant upon a high teacher to student ratio. Although the use of TBL required an instructional approach, needing direction from the tutor, it remained student-centred, generating a range of positive outcomes. In particular, TBL resulted in better pre-class preparation, immediate feedback on progress, and smaller group size. The findings from our study strongly suggest that TBL is the students’ preferred teaching strategy. However, as noted by students during TBL, greater time needs to be devoted to discussion of the pathophysiology flow-chart. Our study results provide confidence for implementing TBL for both Year 1 and Year 2 cohorts within the Sydney Medical Program in 2017.

## Abbreviations

IRAT: Individual readiness assurance test
PBL: Problem based learning
SMP: Sydney Medical Program
SMS: Sydney Medical School
TBL: Team-based learning
TRAT: Team readiness assurance test
